# Pioneering Partnerships: The Irish Model for Embedding the Lived Experience of Dementia in Research and Policy

**DOI:** 10.1002/alz70860_098206

**Published:** 2025-12-23

**Authors:** Cíara O'Reilly, Laura O'Philbin

**Affiliations:** ^1^ The Alzheimer Society of Ireland, Dublin, Ireland; ^2^ Alzheimer Society of Ireland, Dublin, Ireland

## Abstract

Countries adopt varied approaches to Person Public Involvement (PPI) based on cultural, institutional, and policy contexts. The Irish model offers a distinctive example of leading effective engagement. The adoption of PPI as an ethos in Irish dementia research has been bolstered by research institutions, patient organisations and research funders recognising the value of PPI and committing its meaningful inclusion in funding application processes. Targeted national support is provided at an institutional level by PPI Ignite Ireland which promotes excellence and inspires innovation in PPI in Ireland. Integrating PPI features as a key tenet of the Irish Health Research Board's 2021 ‐ 2025 strategy and an essential element of research infrastructure by The Alzheimer Society of Ireland (ASI).

The ASI advocates as a strategic priority for the rights and resources for people living with dementia, family carers and their communities. The organisation grounds its work in the lived experience of dementia and ensures that research and policy across Ireland reflects that experience. In 2019, The ASI established a dedicated PPI panel, the Dementia Research Advisory Team (DRAT), comprising people living with dementia and family care partners. The members of the DRAT (a) influence and inform the research and policy activities of the organisation, and (b) draw on their personal expertise as individuals affected by dementia to collaborate with and advise members of the research community.

This presentation illustrates examples of how the DRAT has successfully collaborated with researchers nationally and internationally to design and implement studies that prioritise relevance, effectiveness and impact. Their work is recognised as an exemplar of how integrating diverse lived experiences enriches the research process.

Ireland's model highlights the importance of structured PPI frameworks, ongoing collaboration, and capacity‐building efforts to empower PPI contributors. By examining Irish PPI initiatives such as the DRAT, who demonstrate a commitment to knowledge exchange through online meetings, workshops and fostering networking opportunities with their research peers, the international research community can gain valuable insights. These insights enable the adaption of similar PPI strategies, promoting a collective practice to further enhance its role in dementia research and policy.

